# Does Surgical Treatment for Benign Prostate Enlargement (BPE)-Related Bladder Outlet Obstruction (BOO) Benefit Patients with Central Nervous System Diseases? A Systematic Review

**DOI:** 10.3390/jcm13195846

**Published:** 2024-09-30

**Authors:** Ioannis Charalampous, Ioannis Tsikopoulos, Calypso Mitkani, Michael Samarinas, Yuhong Yuan, Ioannis Vouros, Petros Tsafrakidis, Anastasiadis Anastasios, Anastasia Gkotsi, Vasileios Sakalis

**Affiliations:** 1 Department of Urology, Hippokrateion Hospital of Thessaloniki, 54642 Thessaloniki, Greece; ioannis.s.charalambous@gmail.com (I.C.); imvouros@hotmail.com (I.V.); 2 Department of Urology, General Hospital of Larisa, 41221 Larisa, Greece; ioannistsikopoulos@yahoo.com (I.T.); mikesamih@hotmail.com (M.S.); 3 Department of Neurology, Agios Pavlos General Hospital of Thessaloniki, 55132 Thessaloniki, Greece; m.calypso11@gmail.com; 4 Division of Gastroenterology, Cochrane UGPD Group, McMaster University, Medicine Health Sciences Center, Hamilton, ON L8S 4L8, Canada; yuhong.yuan@lhsc.on.ca; 5 Germal Oncology Center, Limassol 4108, Cyprus; petertsaf@yahoo.gr; 6 Innovative Surgical and Urological Research Hub (ISUReH), 54250 Thessaloniki, Greece; ngotsi@hotmail.com; 7 1st Department of Urology, Aristotele University of Thessaloniki, 54635 Thessaloniki, Greece; dranastasiadis@yahoo.gr

**Keywords:** prostate surgery, benign prostate enlargement, spinal cord injury, multiple sclerosis, Parkinson’s disease, neurogenic bladder

## Abstract

**Background/Objectives:** Bladder outlet obstruction (BOO) resulting from benign prostate enlargement (BPE) is a common cause of lower urinary tract symptoms (LUTS) in men. Patients with central nervous system (CNS) diseases, such as spinal cord injury (SCI), Parkinson’s disease (PD), cerebrovascular accident (CVA) and multiple systemic atrophy (MSA), commonly experience lower urinary tract dysfunction. Men who suffer from CNS diseases may also experience symptoms related to BPE and BOO, which pose an additional burden to their overall clinical status and result in the need for catheter use and a deterioration in quality of life. The aim of this study was to identify if prostate surgery will benefit men with CNS diseases who have been diagnosed with BPE-related BOO. **Methods:** The systematic review was conducted according to the Preferred Reporting Items for Systematic Reviews and Meta-Analyses (PRISMA) statement. EMBASE, MEDLINE, Cochrane systematic reviews, Cochrane Central Register of Controlled Trials, Google Scholar, and ClinicalTrials.gov were searched from 1946 up to July 2023 for peer-reviewed publications addressing the primary outcome (success rate) and the secondary outcomes (postoperative changes in incontinence episodes, urodynamic parameters, questionnaire scores, and quality of life). In addition, the perioperative outcomes (adverse events and the need for further medical or surgical therapy) were reported. **Results:** A total of 1572 abstracts were screened, and 13 studies involving 1144 patients were eligible for inclusion. Six studies assessed the effect of prostate surgery for BPE-related BOO in SCI, four studies in CVA, two studies in PD, and one study in the MSA population. All studies were considered to have a high risk of bias. Transurethral resection of the prostate (TURP) was the most common de-obstruction procedure, followed by prostatic artery embolism and open prostatectomy. The overall pooled success rate was calculated as 81.4% (65–100%) in SCI, 27.1% (9–70%) in PD, and 66.7% (50–79%) in CVA populations. The risk of de novo incontinence was 24.7–50% in SCI, 20% in PD, 21–50% in CVA, and 60% in MSA population. In patients with SCI with BPE-related BOO, prostate surgery improved mean bladder compliance and detrusor filling pressure and resolved detrusor overactivity in up to 50% of patients. Improvement of free flow rate, voided volume, and post-void residual was observed in all patients. Patients with CVA had an increased risk of perioperative mortality compared to non-CVA patients, and the risk of postoperative complications was inversely proportional to the timing of the CVA insult since surgery. **Conclusions:** This systematic review provides an overview of the available evidence on the outcome of prostate surgery in patients with neurologic diseases and BPE-related BOO. Identifying the optimal practice was challenging due to the limited availability of high-quality studies and the high variability of the reported outcomes. Properly selected patients with neurological diseases may benefit from prostate surgery, provided that preoperative investigations indicate BPE-related BOO.

## 1. Introduction

Bladder outlet obstruction (BOO) resulting from benign prostate enlargement (BPE) is a common cause of lower urinary tract symptoms (LUTS) in men, with a prevalence that increases with age [1]. Benign prostate hyperplasia (BPH), the histological diagnosis that describes the growth of stromal and epithelial cells of the periurethral and transition zones of the prostate gland, is the most common cause of BPE. Epidemiological studies have shown that BPH affects 50% of men in the 60s and almost 90% of men by the age of 80 [2]. BPH is characterized by variable growth of individual cellular composition of the hyperplastic component that range from 16.1% to 56.1% in connective tissues, 20.2% to 59.3% in smooth muscles, 4.3% to 24.8% in the epithelium, and 5.3% to 21.9% in the epithelium lumen [3]. LUTS, which are considered clinical manifestations of BPH, are a heterogenous group of symptoms that are neither disease- nor gender-specific for BOO. LUTS are classified as a storage, voiding, or post-voiding group of symptoms that frequently co-exist [1].

Patients with central nervous system (CNS) diseases frequently present with LUTS. Conditions such as Parkinson’s disease (PD), multiple sclerosis (MS), and spinal cord injury (SCI) lead to a diverse range of lower urinary tract dysfunction (LUTD) and LUTS, depending on the severity and type of the CNS insult. These symptoms can range from urinary incontinence to urinary retention and can exhibit varying degrees of severity, from an indolent course to a highly dangerous state that poses a risk of upper urinary tract deterioration and renal failure. Efforts have been made to estimate the prevalence of neurogenic LUTS; however, it is difficult due to the heterogeneity of the neurogenic population. However, an epidemiological study reported that the prevalence of LUTS in this group ranges from 65.4% to 72.3% and that these patients have a 20% higher risk for the presence of LUTS compared to non-neurogenic participants [4].

Clearly, the risk of BPE-related BOO increases with age and affects all men, including those with CNS diseases, who can also develop LUTS related to BPE. Previous studies have evaluated the prostate volume and serum prostate-specific antigen (PSA) levels of SCI patients, compared them to age-matched individuals, and reported no significant difference, even though SCI men tend to have smaller prostates and lower PSA [5,6]. However, in patients with CNS diseases, the pathophysiology of lower urinary tract dysfunction is far more complex, including detrusor sphincter dysynergia (DSD) as a typical cause of BOO, detrusor muscle overactivity, and impaired bladder compliance [7].

Regardless of the etiology leading to neurogenic bladder, treatment approaches should prioritize the preservation of renal function, improve continence, and enhance quality of life. Managing distressing symptoms such as urinary frequency, incontinence, and incomplete bladder emptying and preventing secondary complications like urinary tract infections (UTIs) are essential in this regard. Furthermore, treatment should focus on keeping patients as catheter-free as possible. In cases where conservative management fails to alleviate symptoms, a surgical approach is typically considered. For men with CNS diseases and BPE-related BOO, prostate-related interventions like transurethral prostatectomy (TUR-P) could potentially alleviate voiding symptoms and improve patients’ quality of life. Because the impact of neurological conditions can cause LUTD at multiple levels, the surgical outcomes for anatomical BOO treatment are anticipated to differ from those in non-neurogenic populations. Thus, postoperative complications such as postoperative urinary incontinence (UI) as well as patient dissatisfaction could be more common in this patient group; a preoperative verification of prostate-related BOO is essential. This is also evident in the non-neurogenic population, as a systematic review has reported the significant relationship between preoperative urodynamic allyproven BOO and better surgical outcomes after TUR-P [8,9].

While prostate surgery is well established for improving outcomes in cases of BPE-related BOO, its efficacy and safety in patients with CNS diseases remain less clear. Given the potential risks and variable outcomes, it is crucial to evaluate whether surgical intervention for BOO in this subgroup of patients is beneficial and, furthermore, to identify which patients might derive the most benefit from such treatment. The potential for postoperative complications, including exacerbation of existing urinary symptoms or the development of new urological issues, must be carefully weighed against the expected benefits of BOO treatment. Factors such as cognitive impairment, mobility limitations, and progression of the underlying neurological disease should be taken into account when considering surgical options. Additionally, the choice of anesthesia and perioperative management strategies may need to be modified to accommodate the specific needs of patients with CNS disorders. A previous Cochrane study conducted a comprehensive evaluation of the management of BOO secondary to DSD, while a recent systematic review assessed the surgical management of anatomic BOO in neurologic patients, including BPO and urethral strictures [7,10]. Hence, we identified a gap in the literature and aimed to assess postoperative outcomes following surgery for BPE-related BOO in men with CNS diseases and neurogenic bladder dysfunction caused by conditions such as spinal cord injury (SCI), Parkinson’s disease (PD), cerebrovascular accident (CVA), and multiple system atrophy (MSA).

## 2. Materials and Methods

### 2.1. Search Strategy

The databases of EMBASE, MEDLINE, Cochrane systematic reviews, Cochrane Central Register of Controlled Trials (CENTRAL; Cochrane HTA, DARE, and HEED), Google Scholar, and ClinicalTrials.gov were searched from 1946 up to July 2023. The study protocol was published on PROSPERO (CRD42023463532). Only English-language articles published from 1974 onwards were included. The detailed search strategy is described in the Supplementary Material. Additional sources for articles included the reference lists of the included studies as well as systematic and literature reviews. Two reviewers (I. C. and I. T.) screened all abstracts and full-text articles independently. Any potential conflicts or queries were independently reviewed by a senior author (V. S.) who acted as an arbiter. Data extraction was performed by the same reviewers and corroborated by the senior author.

### 2.2. Types of Study Design

All types of peer-reviewed publications addressing the primary and secondary outcomes after prostate surgery for BPE in men with neurogenic bladder were eligible. The minimum cohort size was 5 men. Conference abstracts, case reports, and non-English language publications were excluded.

### 2.3. Types of Participants

The study population included men with neurogenic lower urinary tract dysfunction who have had any intervention on prostate for urodynamic allyproven or clinically presumed bladder outlet obstruction due to BPE. All men diagnosed with any type of urological malignancy involving either the bladder, prostate, urethra, or penis were excluded. In addition, men with a history of kidney or testicular malignancy or other systemic malignancies were excluded if they underwent prostate surgery within 5 years from diagnosis.

### 2.4. Types of Intervention

All surgical treatment options for the management of BPE were included. Potential interventions included resection techniques (monopolar/bipolar transurethral resection of prostate, laser vapore section, and transurethral incision of prostate), enucleation (open prostatectomy, bipolar enucleation, and laser enucleation), vaporization (bipolar and laser prostate vaporization), alternative ablative techniques (aquablation, prostate artery embolization, and convective water vapor energy ablation), and non-ablative techniques (prostatic urethral lift, intraprostatic injections, and iTIND). For these techniques, any pairwise comparisons were allowed (including no comparator).

### 2.5. Outcome Measures

The primary outcome was the success rate of the intervention. Considering the anticipated variability across studies in terms of outcome reporting, the interpretation of success was determined by individual trialists. The secondary outcomes were the following: postoperative changes in continence status, postoperative changes in urodynamic parameters, changes in questionnaire scores, and changes in quality of life. In addition, the perioperative outcomes (adverse events and the need for further medical or surgical therapy) were reported. The results are presented as a pooled mean and percentage values.

### 2.6. Assessment of Risk of Bias

The risk of bias (RoB) of each included study was assessed by the two review authors working independently. Potential disagreements were resolved by the senior author. The RoB of non-randomized comparative studies was assessed using the Cochrane tool ROBINS-I, including additional items to assess the risk of confounding bias [11]. This included assessment of the following seven domains: random sequence generation, allocation concealment, blinding of participants and personnel, blinding of outcome assessment, incomplete outcome data, selective reporting, and other sources of bias. Each study was rated as “low”, “high”, or “unclear” [12]. The following confounders were identified a priori: documented BOO due to BPE, age, and prostate volume. For case series, the five-criterion quality appraisal checklist was used, as recommended by the European Association of Urology Methodology group [13]. The checklist included the following questions: Was there an a priori protocol? Was the total population included, or were study participants selected consecutively? Was outcome data complete for all participants, and was any missing data adequately explained/unlikely to be related to the outcome? Were all prespecified outcomes of interest and expected outcomes reported? Were primary benefit and harm outcomes appropriately measured? If the answer to all five questions was “yes”, the study was assessed as “low” RoB. If the answer to any question was “no”, the study was considered “high” RoB.

### 2.7. Data Analysis

Due to the lack of RCTs, meta-analysis and quantitative analysis were not appropriate, so a narrative synthesis approach was used instead. Although a subgroup analysis was initially planned, it was not possible due to the inadequate number of studies. Therefore, a narrative review of outcomes was performed.

## 3. Results

### 3.1. Quantity of Evidence Identified

**Figure 1:**
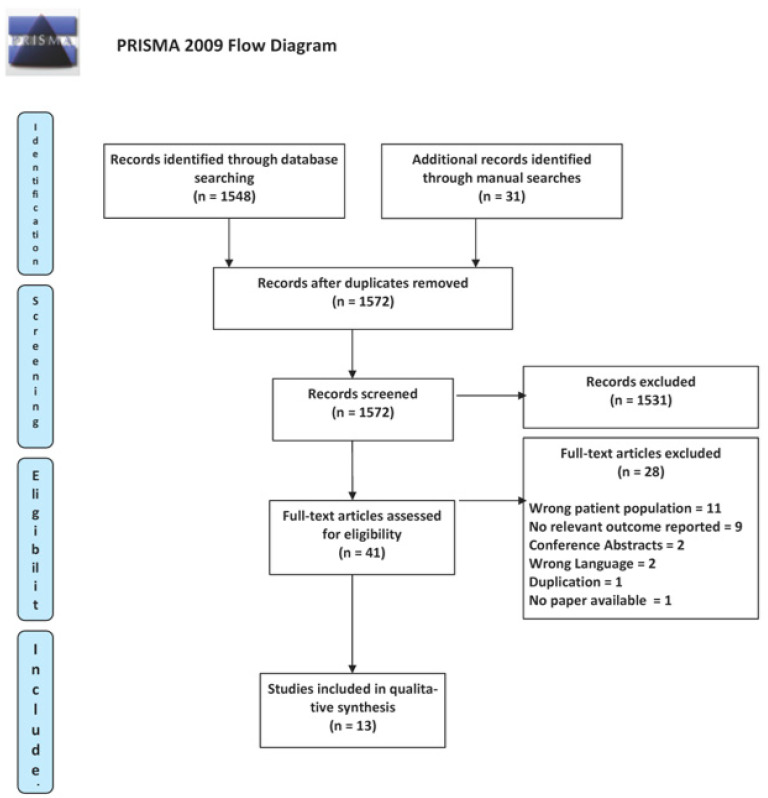
The PRISMA flow diagram of the study.

A total of 1572 abstracts were screened, and 41 studies were retrieved for full-text screening. A total of 13 studies were eligible for inclusion involving 1144 patients: 11 case series (536 patients) [14,15,16,17,18,19,20,21,22,23,24] and 2 NRCSs (608 patients) [25,26] [Figure 1].

### 3.2. Characteristics of the Included Studies

The baseline characteristics of the included studies are presented in Table 1. All studies were retrospective. The two NRCS assessed the effect of prostate surgery in patients with neurogenic LUTD due to CVA [25,26]. The eleven cohort series assessed the effect of prostate surgery in patients with SCI (six studies) [15,16,17,19,20,23], CVA (two studies) [22,24], Parkinson’s disease (two studies) [14,18], and multiple systemic atrophy (one study) [21].

### 3.3. RoB and Confounding Assessment of the Included Studies

**Figure 2:**
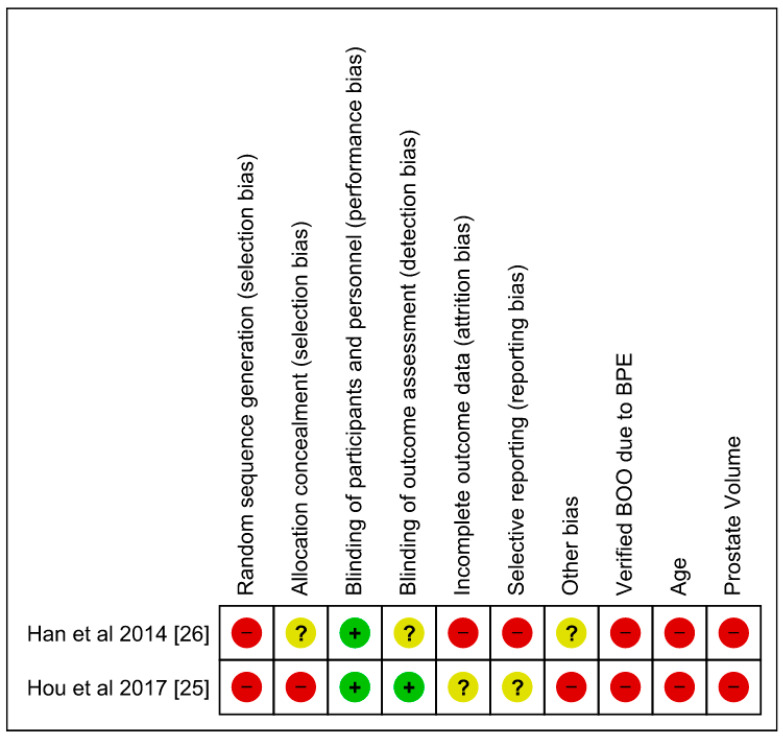
Risk of bias summary.

**Figure 3:**
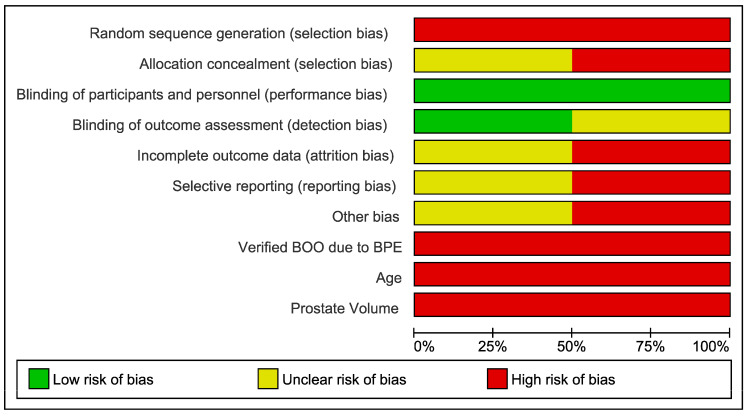
Risk of bias graph.

The RoB assessments for the NRCT are summarized in Figure 2 and Figure 3. NRCTs were considered high RoB. For the majority of the studies, selection, performance, detection and attrition biases were considered high, while reporting biases were considered unclear or high. All case series were high RoB.

### 3.4. Narrative Synthesis of the Results

#### 3.4.1. Description of Population Characteristics from the Included Studies

##### Spinal Cord Injury Population

Data regarding men suffering from SCI and undergoing any type of prostate intervention came from six case series [15,16,17,19,20,23] involving 312 patients. All studies were classified as having a high risk of bias. In five studies [15,17,19,20,23], TURP was the specified intervention, while in the remaining study [16], prostate embolism was the intervention of choice. In three studies [17,19,20], TURP was characterized as a radical that included the resection of the peripheral zone down to the anatomical capsule. Five studies reported on the level of SCI in 277 patients [15,16,17,19,20]. The mean age of patients was 56.7 years, with a range of 20–86 years. The follow-up ranged from 6 months to 11 years. A total of 97 men (35%) had cervical SCI, 94 men (34%) had thoracic SCI, and 86 men (31%) had lumbar/sacral SCI.

The most typical symptoms that prompted patients to seek care and undergo surgery were voiding-type LUTS. Data from four studies and 200 patients showed that most patients used catheters either in the form of indwelling (157/200 (78.5%)) or intermittent self-catheterization (14/200 (7%) [16,17,19,20]. Three studies used pressure-flow studies [17,19,20] and one used video urodynamics [14] to diagnose BPE-related BOO.

##### Parkinson’s Disease Population

Three studies provided data on male patients diagnosed with PD who had undergone prostate intervention [14,18,24]. Two studies [14,18] assessed PD patients only, while the third [24] included patients with other neurological insults as well, with PD patients comprising 17% of the study population. The outcomes of 70 men were analyzed herein. All studies were classified as having a high risk of bias. In all three studies, men undertook TUP-P or TUR incision of the prostate. One study included patients classified as Hoehn and Yarh ≤3 [14], while another one [24] included patients with vascular parkinsonism (36%) and multiple system atrophy (9%). The mean age of patients was 70.3 years, with a range of 50–82 years. The follow-up ranged from 1 to 60 months.

The most common symptoms that led patients to seek treatment and opt for surgery were storage-type LUTS, including urgency, frequency, and urge incontinence. One study [14] reported that 14/23 (61%) of patients used catheters, and the remaining 9/23 (39%) were voiding with control. Pressure-flow study, gas cystometry, and video urodynamics were used to assess BPE-related BOO.

##### Post-Cerebrovascular Accident Population

Four studies provided data on male patients with LUTS and a history of CVA who had undergone prostate intervention [22,24,25,26]. Three studies [22,25,26] assessed post-CVA patients only, while one [24] included a mixed population with the post-CVA patients comprising 68.7% of the study sample. This review analyzed the outcomes of 691 men. All studies were classified as having a high risk of bias. All men undertook TUR-P except a few patients who had open prostatectomy (7/691, 1%). The mean age of patients was 72.5 years, with a range of 62–84 years. The follow-up ranged from 3 to 60 months.

The most common symptoms that led patients to seek treatment and opt for surgery were mixed-type LUTS, predominantly voiding symptoms and urinary retention. None of the included studies provided information on preoperative bladder management. Uroflowmetry was used to diagnose obstruction in one study [26], while pressure-flow studies or video urodynamics were used in the other two [22,24]. One study did not report any relevant information [25].

##### Multiple Systemic Atrophy Population

One study assessed the outcomes of prostate surgery in the MSA population [21]. This was a mixed-gender study, but the data of the 20 men who underwent TURP was available. The study was considered to have a high risk of bias. The symptoms that led patients to seek treatment were voiding LUTS, while BPE-related BOO was diagnosed clinically.

#### 3.4.2. Primary Outcome

The primary outcome was defined as the success rate after intervention (Table 2).

##### Spinal Cord Injury Population

The definition of successful outcome varied among the included trials. Two trials used subjective criteria such as patient satisfaction [15] and voiding improvement [17], while the remaining used objective findings such as easy catheterization [16], no need for retreatment [19], being catheter-free [20], and reduced post-void urine residual [23]. The overall pooled success rate was 81.4%, as 254/312 cases were considered successful. The success rate ranged from 65% to 100%.

##### Parkinson’s Disease Population

A successful outcome was considered to be the improvement of symptoms [18,24] and change in bladder management [14]. The overall pooled success rate was 27.1%, as 19/70 cases were considered successful. The success rate ranged from 9% to 70%.

##### Post-Cerebrovascular Accident Population

According to the studies included, success was defined as symptomatic, including reduction of acute urinary retention (AUR) episodes. However, only two trials provided adequate data [22,24]. The overall pooled success rate was 66.7%, as 52/78 cases were considered successful. The success rate ranged from 50% to 79%. In addition, data for post-TURP voiding improvement were extracted from two studies based on the AUR episodes. In one study, retention was resolved in 89% of men (16/18) after TUR-P [24], while in the second, the percentage of AUR episodes at 12 months after TURP reduced by 33% [25]. The comparison between ischemic vs. hemorrhagic CVA in reducing post-TURP AUR episodes at 12 months was −32.3% vs. −36.7% [25].

#### 3.4.3. Secondary Outcomes

Data regarding secondary outcomes were not reported in all studies. The available evidence is described below as per the technique used.

##### Postoperative Change in Continence

Data on postoperative change in continence was available in six trials [14,15,17,18,22,24]. Two studies reported the post-TURP changes in continence status in patients with SCI [15,17]. The risk of de novo UI was calculated as being between 24.7% and 50%. According to one study, 25/42 (59.5%) reported that their urinary incontinence (UI) did not improve or worsen, while in 16/42 (38.1%), continence was improved. Only 2.4% of participants reported a worsening of their UI [17].

Three studies reported changes in continence status after prostate surgery in patients with PD [14,18,24]. Before intervention, 27 patients presented with urge UI. Postoperatively, the incontinence was resolved in 7/27 (25.9%), improved in 3/27 (11.1%), and remained unchanged in 15/27 (55.6%) patients. One study reported the occurrence of de novo urge UI in 6/30 (20%) previously continent men [18].

In the CVA population, prostate surgery worsened continence in 21–50% of patients [22,24], with urge UI being more troublesome. The timing of the operation in relation to CVA has been correlated to the outcome. The majority of patients who were operated on at least 12 months after CVA had better continence status compared to those who had been surgery earlier than 12 months (76.5% vs. 23.5%, *p* < 0.01) [22]. The study on MSA reported that 12/20 (60%) of men developed de novo UI after prostate surgery [21].

##### Postoperative Change in Urodynamic Parameters

Data on postoperative changes in urodynamic parameters were available in four trials [14,17,19,20,24]. In the majority of them, urodynamics took place in only those who voided spontaneously.

Three studies reported on the post-TURP changes in the SCI population [17,19,20]. There was a significant increase in mean bladder compliance (+18.3 mL/cmH2O, *p* < 0.05), a significant reduction in detrusor filling pressure (−18.7 cmH_2_O), and a resolution of detrusor overactivity in up to 50% of patients [17,19]. Three studies reported post-TURP improvement of flow rate and PVR (post-void residual), which were +11 mL/s and −27.6 mL, respectively [17,19,20]. TURP appeared to benefit men with vesicoureteral reflux, as a resolution up to 65% and an improvement up to 13% were noted [19].

Two studies provided data on changes in parameters after TURP in men with PD [14,24]. Both studies reported improvement in the flow rate (+1.7 and +10 mL/s) and voided volume (−19 and +220 mL). One study reported a significant improvement in voiding efficiency (+0.11, *p* = 0.04) [24]. In men with CVA, there was a significant improvement in PVR (−63.4 mL, *p* = 0.044) and voiding efficiency (+0.19, *p* = 0.004) [24].

##### Postoperative Changes in Questionnaires and Quality of Life Scores

Data on postoperative change in relevant questionnaire scores were available in two trials [14,15]. A study on the SCI population reported that 86.5% of men were satisfied after TURP; however, no official questionnaires were used [15]. On the contrary, authors in a study on the PD population used the International Prostate Symptom Score (IPSS) and reported improvement of −12 (*p* = 0.028) in the total IPSS score and −2(*p* = 0.026) in the quality of life score [14].

##### Perioperative Complications

Five studies reported on perioperative complications, but none used a formal grading system [15,16,19,23,25]. A retrospective study on the SCI population reported a 15.6% risk of autonomic dysreflexia, an 82% risk of urinary tract infections, and a 59% risk of difficult urination [15]. Patients with a history of CVA also had a higher risk of perioperative complications. The adjusted odds ratio (OR) was calculated as 1.37(95%CI: 1.13–1.68, *p* = 0.002) to develop UTI up to 12 months postoperatively and 1.89(95% CI: 1.51–2.36, *p* < 0.001) to develop urinary retention requiring catheterization [25]. This study also reported the difference in perioperative mortality between CVA and non-CVA patients (1% vs. 0.1%, *p* = 0.001) with an adjusted OR of 8.27(95%CI: 2.47–27.7, *p* = 0.001) [25].

A comparative study on the CVA population reported a significant difference in perioperative mortality between CVA and non-CVA patients (1% vs. 0.1%, *p* = 0.001) [25].

##### Need for Additional Medical or Surgical Therapy

Five studies reported on the need for additional medical or surgical therapy after prostate surgery to control LUTS [15,16,19,23,25]. In the SCI population, 31% (27/87) required postoperative pharmacotherapy, such as anticholinergics or a-blockers [15,16]. Moreover, 27.2% (22/81) of men required prostate re-operation, while a remarkable percentage of patients required other procedures such as botulinum toxin injections [15,16,19,23]. A history of CVA did not increase the risk of prostate re-operation (4.2% vs.6.9%, *p* = 0.061) [25].

## 4. Discussion

The primary objectives of neurogenic lower urinary tract dysfunction management are to preserve upper urinary tract function, minimize complications arising from the lower urinary tract, improve continence, enhance patients’ quality of life, and limit the use of diapers or other collection devices. Managing neurogenic lower urinary tract dysfunction can be a challenging task, as it is often difficult to achieve all these objectives simultaneously.

Our findings suggest that properly selected patients with neurological diseases may benefit from prostate surgery, provided that preoperative investigations indicate BPE-related BOO. Albeit the variability in the definition of success among the included trials, it is evident that prostate surgery has a role in the management of these patients, especially in men who present with LUTS that are linked to both neurological dysfunction and prostatic enlargement. The reported overall success rate was 81.4% in SCI, 27.1% in PD, and 66.7% in CVA populations. Furthermore, despite the lack of high-level evidence, preoperative urodynamic evaluation is essential to differentiate between BPE-related BOO and other causes of voiding LUTS, ensuring that surgical interventions target the appropriate pathology [1]. Despite the availability of various treatment options, there are limitations to the current management strategies that must be taken into consideration.

Advancements in the care of SCI patients have led to a notable increase in life expectancy. Men of higher age may be troubled by BPE-related complications to the same extent as neurologically intact individuals. There is evidence that prostate size and PSA serum levels are slightly lower in the SCI population, although the differences are not significant [5]. BPE could present as hematuria, obstructive voiding, difficult catheterization, urinary tract infections, etc. Especially for obstructive voiding and urodynamically proven BOO, it is difficult to differentiate between BPE and DSD in the SCI population. Despite the challenges, accurate diagnosis of BPE and DSD in patients with SCI and obstructive voiding is crucial for effective treatment and management. The success rate after TUR-P in this population was 81.4% (65–100%), which indicates that the procedure is effective in managing symptoms and improving patient outcomes. While the criteria of success may vary among studies, it is important to consider both objective and subjective findings when defining efficacy. Evidence from urodynamic data suggests that TUR-P improves flowrate (+11 mL/s) and PVR (−27.6 mL/s), which is comparable to neurologically intact individuals [1]. Our research findings align with the conclusions of a prior systematic review that suggests that TUR-P may be a viable option in SCI men with BPE-related BOO [10].

However, the surgical technique differs between the included trials. Three studies have evaluated the role of radical TUR-P in patients with SCI and BPE-related BOO that was refractory to conservative therapy LUTS [17,19,20]. Interestingly, the postoperative success rate was reported as 90%, 86.5%, and 92%, respectively. This modified TUR-P technique involves the resection of the prostate, including the peripheral zone, and extending down to the true prostatic capsule of both sides of verumontanum. The initial concept was that radical TUR-P could be devised as surgical sympathectomy because the technique removes the adrenergic system from the region of the external urethral sphincter in the manner of an intramural sympathectomy [19]. The authors suggested that radical TUR-P could potentially relieve DSD by suppressing or abolishing abnormal continence reflex, while continence is preserved by the activity of the untouched external urethral sphincter [17].

An undesirable consequence following prostate surgery in SCI patients is the potential risk of de novo or worsening of pre-existing UI. We found a high risk of de novo UI of 24.7–50%, which is much higher than previously reported [10]. The discrepancy is due to the inclusion of a newly introduced study that was previously unavailable [15]. Up to a third of SCI patients after BPE surgery will need further additional medical or surgical therapy to manage overactive bladder symptoms. This indicates that de novo UI might result from remodeling of bladder neural pathways, urethral pooling in the prostatic fossa activating the micturition reflex, or by another pathophysiologic mechanism that remains to be elucidated. Postoperative urodynamic data suggest that bladder compliance improves, detrusor overactivity resolves, and pre-existing vesicoureteral reflux resolves in up to 65%.

Evidence from the literature suggests that SCI patients experience significantly higher complication rates compared to neurologically intact subjects. Gamon et al. reported that 57% of SCI men experienced complications after radical prostatectomy, a number that is far higher, even when comparing low-volume surgeon complication rates [27,28]. However, radical prostatectomy is a major operation and far more complex compared to BPE interventions. Comparative data of complication rate after BPE surgery in SCI patients and an age-matched healthy population is missing from the literature. An explanation is that patients with SCI are younger than the neurologically intact patients who suffer from BPH, making it impossible to conduct a direct comparison between the two groups. Evidence from a single study reported that after TUR-P, there was an 82% risk of urinary tract infections and a 59% risk of difficult urination [15]. A systematic review on the complications of transurethral procedures for LUTS due to BPE in neurologically intact men reported a 0–22% risk of postoperative urinary tract infections and a 0–13.3% risk of urinary retention [29]. The reported risk of autonomic dysreflexia after TURP was 15.6%, which was higher compared to the 8% reported after radical prostatectomy or cysto-prostatectomy [15,27]. The justification for this difference is twofold. The first is the small size of the study population. It was not sufficiently powered, resulting in type II statistical error, overestimation of effect, and false conclusions. The second is the level of injury. Tetraplegics were 29% and 56% in the studies of Gammon et al. and Wu et al., respectively, while the percentage of patients with thoracic SCI was 43% and 17%, respectively.

Parkinson’s disease (PD) is a neurodegenerative disorder that affects 2–3% of the population aged >65 years [30]. The pathophysiological hallmark of PD is neuronal loss in the substantia nigra that causes striatal dopamine deficiency and intracellular inclusions containing aggregates of a-synuclein. This loss of dopamine in the striatum leads to the characteristic motor symptoms of PD, such as bradykinesia, rigidity, and resting tremor. Additionally, this loss of dopamine also contributes to non-motor symptoms, such as depression, anxiety, and cognitive decline, which are commonly experienced by individuals with PD [31]. Furthermore, these non-motor symptoms can significantly impact the quality of life for individuals with PD. The majority of patients exhibit a degree of autonomic dysfunction, such as orthostatic hypotension, urogenital dysfunction, and constipation. A recent systematic review has reported that the prevalence of LUTS in PD patients was 61%, while the pooled prevalence of storage and voiding symptoms was 59% and 24%, respectively [32].

A careful examination of the literature reveals that the success rate of TUR-P has improved over the years in the PD population. The traditional concerns surrounding poor safety and low efficacy appear to have vanished as new evidence has emerged. Previous studies suggesting that individuals with PD were not suitable for prostate surgery may have been misleading, as earlier research included patients with multiple system atrophy who were mistakenly diagnosed with PD. Multiple system atrophy (MSA) is a rare alpha-synucleinopathy characterized by a combination of prominent autonomic dysfunction with extrapyramidal signs and/or cerebellar ataxia [9]. However, more than half of MSA patients present with LUTS even before the manifestation of motor symptoms [33]. In these patients, detrusor contractility is often impaired, resulting in urinary retention, de novo incontinence, and a frequently unfavorable postoperative outcome.

Our review included three studies in the PD population, published in 1988, 2009, and 2023 [14,18,24]. The overall success rate was calculated as 27.1% (9–70%). This is partly due to sample bias but also due to the remaining troublesome storage symptoms. However, only Roth et al. reported that MSA patients had been excluded from the analysis and that the success rate of the PD population was 70% (16/23) at the three-year follow-up [14]. The pooled evidence showed that after prostate surgery, 55.6% of patients maintained their continence, and it was rather improved (11.1%) or even resolved (25.9%). The prevalence of de novo urinary incontinence was 20% and has been documented only in prior research involving older adults, which may have included individuals with PD and MSA [18]. The improvement following TUR-P has been verified objectively by demonstrating a substantial increase in free flow rate and voiding volume. Furthermore, it has been confirmed subjectively through a notable improvement in total IPSS and quality of life scores.

Cerebrovascular accidents are more common in patients aged >65 years, with a prevalence of 4.6–7.3%. Strokes are associated with significant disability due to motor impairment, loss of independence, depression, and voiding dysfunction [34]. Post-CVA LUTS is a prevalent issue that affects a substantial number of patients. The most common pattern of micturition disturbance following a stroke is urinary retention preceded by resolution or development of urge urinary incontinence [35]. While the majority of patients demonstrate detrusor overactivity, a study identified a relationship between chronic pontine stroke and detrusor underactivity [35,36]. Individuals who have experienced CVA often suffer from conditions that can influence the function of the lower urinary tract (LUT), including diabetes mellitus [37]. In this report, we have documented an overall success rate of 66.7% (50–79%) for patients who underwent TURP after experiencing a CVA. Success was determined by the decrease in the number of urinary retention episodes. However, the risk of incontinence after TUR-P is not negligible, and it is directly related to the extent of neurologic impairment as well as the timing of the operation [22].

Patients with a history of CVA are at high surgical and anesthetic risk. CVA is the most common medical condition requiring intensive care unit admission [38]. The medical profile of these patients frequently includes several risk factors, such as diabetes mellitus, hypertension, arrythmias, and chronic kidney diseases, as well as advanced age, as the majority of CVA patients are between 61 and 80 years old [37]. The study by Hou et al., which provided data on the perioperative adverse events after prostate surgery in these patients, stated that heart failure, ischemic heart disease, and Parkinson’s disease were more prevalent in the CVA group and that a higher amount of prostate tissue was removed, a factor that could influence perioperative morbidity [25]. The presence of these comorbidities, along with the greater extent of prostate tissue removal, highlights the importance of careful patient selection and perioperative management to minimize the risk of adverse events in patients undergoing prostate surgery.

According to the results presented above, it is clear that BPE-related BOO can negatively impact the quality of life for individuals with CNS diseases. Therefore, in cases where conservative treatment options have proven unsuccessful, prostate surgery may be a viable therapeutic option for select patients. This study’s strengths are rooted in the systematic and transparent approach, the pre-existing protocol, and adherence to the Preferred Reporting Items for Systematic Reviews and Meta-Analyses (PRISMA) guidelines. The limitations of this review are strongly related to the base of evidence that was analyzed. All the included studies were retrospective cohort series that were considered high-risk. With the exception of two studies involving CVA patients [25,26], the included studies assessed a limited number of subjects, which may have influenced the research outcomes and potentially compromised the validity of the studies. Moreover, considerable variability in terms of patient characteristics, diagnosis of BOO, technique of prostatectomy, and reported outcomes was encountered. To address these limitations, the results were reported separately for each neurological condition, presenting the number of successful cases in relation to the total number of cases as well as providing the range of success rates. The available evidence is insufficient to draw definitive conclusions regarding the efficacy of prostate surgery for patients with CNS diseases. The paucity of conclusive data precludes a comprehensive assessment of potential improvements or benefits that such patients might derive from prostate surgical interventions. Further research is warranted to elucidate this complex clinical question and establish a more robust evidence base.

In addition, it is important to use standardized terminologies and definitions of outcomes in accordance with the International Continence Society’s guidelines [39]. Additionally, future studies could focus on specific patient groups comparing specific interventions to investigate long-term efficacy, health outcomes, and potential health-related costs.

## 5. Conclusions

We have systematically reviewed available studies assessing the effect of prostate surgery in patients with central nervous system diseases and BPE-related BOO. The findings and clinical relevance were interpreted using an appropriate clinical context provided by a panel of urologists and neurologists. There is evidence to support that prostate surgery could benefit selected patients suffering from SCI, PD, and CVA, provided that preoperative investigations indicate BPE-related BOO. Limitations include the current evidence base, the lack of large prospective studies, and the lack of RCTs or comparative trials. Despite these limitations, this review provides up-to-date evidence of the predefined variables that can help to provide clinical guidance, although it has not identified the optimal practice in surgical treatment.

## Figures and Tables

**Table 1:** Baseline characteristics of the included studies.

Data Extraction Sheet												
Study ID	Study Design Period	Included NU Patients/Study Population	Type of NU Patients (%)	Type of Intervention (%)	Patient Age (Mean, yr)	Type of NLUTD (Symptoms %)	Indication for Surgery	Preoperative Bladder Management	Preoperative Incontinence (%)	Follow-up Time	Risk of Bias	
Roth et al. (2009) [14]	Retrospective, 1997–2007	23/23 (100%)	23/23(100%) Parkinson’s disease (Hoehn and Yarh scale ≤3)	TURP 23/23 (100%)	Median 73 (IQR: 68–81)	Voiding (23/23) 100%, Urge Incontinence (10/23) 43%	LUTS refractory to medical therapy	IDC: 14/23 (61%) VWC: 9/23 (39%)	UUI: 10/23 (43%)	Median 3 yr (IQR 2–6)	High Risk	
Wu et al. (2022) [15]	Retrospective, 1997–2020	261/261 *	77/77 (100%) SCI Cervical SCI: 43/77 (56%) Thoracic SCI: 13/77 (17%) Lumbar/sacral SCI: 21/77 (27%)	TUIP/TURP 77/77 (100%)	NR	NR	LUTS refractory to medical therapy	NR	0%	Median 11 years (IQR 6.17)	High Risk	
Sampogna et al. (2022) [16]	Retrospective, 2015–2020	10/10 (100%)	10/10 (100%) SCI Cervical SCI: 5/10 (50%) Thoracic/sacral SCI: 5/10 (50%)	Prostatic Artery Embolization (PAE) 10/10 (100%)	Median 70 (Range:59–86)	Urinary retention, BOO	Difficulties in performing ISC due to BPH 10/10 (100%)	IDC: 7/10 (7%) ISC: 3/10 (30%)	0%	Median 33 months	High Risk	
Shino et al. (1994) [17]	Retrospective, 1977–1989	51/51 (100%)	51/51 (100%) SCI Cervical SCI: 8/51 (16%) Thoracic SCI: 25/51 (49%) Lumbar/sacral SCI: 18/51 (35%)	TURP* Radical TUR-P that includes the resection of the peripheral zone down to the anatomical capsule	Mean: 49.2 (Range: 22–73)	Voiding 51/51: (100%), Incontinence: 42/51 (82%) * Mild UI: 14/42 (33.3%) * Moderate UI: 22/42 (52.4%) * Severe UI: 6/42 (14.3%)	LUTS refractory to medical therapy	VWC: 29/51 (57%) ISC: 11/51 (21.5%) IDC: 11/51 (21.5%)	UI: 42/51 (82%) * Not specified type of incontinence	NR	High Risk	
Stanskin et al. (1988) [18]	Retrospective, 1977–1984	36/50 (72%)	36/36 (100% Parkinson’s disease)	TURP36/36 (100%)	NR (entire population mean 67, range 50–82	92% OAB, 8% acontrile detrusor (entire population)	BOO	NR	UI 6/36 (17%) * UUI: 4/6 (67%) * Overflow UI: 2/6 (33%)	Median 9.2 months (1 to 28 months)	High Risk	
Koyanagi et al. (1987) [19]	Retrospective, 1981–1986	89/89 (100%)	89/89 (100%) SCI Cervical SCI: 20/89 (22.5%) Thoracic SCI: 34/89 (38%) Lumbar/sacral SCI: 35/89 (39.5%)	89/89 TURP*Radical TUR-P includes the resection of the peripheral zone down to the anatomical capsule	Mean: 50 (Range: 20–80)	Voiding 89/89 (100%), due to DSD	Voiding LUTS	IDC: 89/89 (100%)	0/89 (100%)	Mean follow-up of ~4 years	High Risk	Same group as Shinno et al.
Koyanagi et al. (1981) [20]	Retrospective, NR	50/50 (100%)	50/50 (100%) SCI Cervical SCI: 12/50 (24%) Thoracic SCI: 20/50 (40%) Lumbar/sacral SCI: 18/50 (36%)	59/50 TURP*Radical TUR-P that includes the resection of the peripheral zone down to the anatomical capsule	NR	Voiding 50/50(100%)	Unsatisfactory voiding function defined as PVR >50 mL and Qmax <5 mL/s and detrusor pressure during voiding <60 cmH_2_0. Additionally, patients with SCI and worsening kidney function	IDC: 50/50 (100%)	0/50 (0%)	Longest 5 years, shortest 6 months; mean 2.5 years	High Risk	
Beck et al. (1994) [21]	Retrospective, 1987–1994	46/62 (100%)	62/62 (100%) Multiple system atrophy	20/46 (44%) TUR P	NR	Voiding suggecting BOO	Voiding LUTS	NR	0/20 (0%)	NR	High Risk	
Hou et al. (2017) [25]	Retrospective comparative study, 1997–2012	577/6625 (8.7%)	577/577 (100%) CVA patients	TURP	CVA: 74.1 ± 7.9 vs. non-CVA: 71.9 ± 8.3	UTIs within 3 months: 27.7% (160/577) vs. 18.4% (1112/6048) AUR: 47.7% (275/577) vs. 34.3% (2075/6048)	AUR, UTIs	NR	NR	5.0± 3.8 vs. 6.9 ± 4.4 years	High Risk	
Han et al. (2014) [26]	Retrospective comparative study, 2009–2011	31/372 (8%)	31/31 (100%) CVA	31/31 (100%) TURP	NR	Urinary retention, BOO,		Medications	0%	3 months	High Risk	
Lum et al. (1982) [22]	Retrospective, 1975–1979	39/39 (100%)	39/39 (100%) CVA	32/39 (94.1%) TURP, 7/39 (5.9%) open prostatectomy	72.4	AUR, chronic retention, prostatism, and incontinence	Acute and chronic urinary retention, prostatism, and incontinence	NR	NR	11–15 months	High Risk	
Elsaesser and Stoephasius (1972) [23]	Retrospective, 1969–1971	35/46 (76.1%)	SCI	21/35 (60%) TURP, 14/35 (40%) Bladder neck incision	NR	NR	To improve voiding	NR	NR	NR	High Risk	
Chang et al. (2022) [24]	Retrospective, NR	64/64 (100%)	44/64 (68.7%): CVA 11/64 (17.2%)-Parkison’s disease, 8/64 (12.5%): early-stage demetia	TURP, TUI-P, TUI-BN	71.1 ± 9.8	OAB, BOO refractory to medical treatment, nocturia, urgency, and urinary incontinence	BOO	NR	37/64 (58.7%)	Median 2 years (6–60 months)	High Risk	
Chang et al. (2022) [24]	Retrospective, NR	64/64 (100%)	44/64 (68.7%): CVA 11/64 (17.2%)-Parkison’s disease, 8/64 (12.5%): early-stage demetia	TURP, TUI-P, TUI-BN	71.1 ± 9.8	OAB, BOO refractory to medical treatment, nocturia, urgency, and urinary incontinence	BOO	NR	37/64 (58.7%)	Median 2 years (6–60 months)	High Risk	

* The analysis included 77 men who underwent TURP-TUIP. The study included patients who had TUI-BN with mixed popluation, and it was therefore excluded to avoid result contamination.

**Table 2:** Primary and secondary outcome scores of the included studies.

Data Extraction Sheet									
Study ID	Success (Defined as per Trialist)	Change in Incontinence Episodes After Surgery	Change in Urodynamic Parameters after Surgery	Change in Questionaire Scores	Change in QoL	Changes(Catheters Before/After, Pads Before/After, etc.)	Adverse Effects After Surgery	Need for Reoperation–Reintervention	
Roth et al. (2009) [14]	Definition of success: improvement of voiding parameters and change in bladder management 16/23 (70%)	No episodes of de novo UI 5/10 men with UUI restored continence (50%) 3/10 men with UUI had improved continence (30%)	Qmax: +10 mL/s (*p* = 0.028) * VV: +220 mL (*p* = 0.018) #	IPSS total score: −12 (*p* = 0.028) #	IPSS QoL score: −2 (*p* = 0.026) #	9/14 (64%) restored voiding. 1/14 (7%) on IDC 4/14 (29%) on ISC	NR	NR	
Wu et al. (2022) [15]	Definition of success: patient satisfaction 50/77 (65%) Cervical SCI: 29/43 (67.4%) Thoracic SCI: 9/13 (69.2%) Lumbar/sacral SCI: 12/21 (57.1%)	UI (UUI/SUI) 19/77 (24.7%)	NR	NR	86.5% satisfied, no official questionnaires	NR	Recurrent UTIs 65/77 (82%) Difficult Urination 41/77 (59%) UI (UUI/SUI) 19/77 (24.7%). Autonomic dysreflexia 12/77 (15.6%)	34/77 (44.2%) Botox 9/24 (37.5%) TUI-BN: 4/24 (16.7%) Re-TUIP/TURP: 11/24 (45.8%)	
Sampogna et al. (2022) [16]	Definition of success: * Easy ISC 10/10 (100%) * Technical success (bilateral embolization) 9/10 (100%)	NR	NR	NR	NR	ISC: 10/10 (100%)	none	0/10 (0%) for BPH 1/10 (10%) Botox to external urethral sphincter	
Shino et al. (1994) [17]	Definition of success: * Subjective or objective improvement of voiding: 47/51 (90%) * UI improved or resolved: 16/42 (38%)	4/9 men developed de novo SUI (45%). 4/42 men with UI restored continence (10%) 12/42 men with UI had improved continence (29%) 25/42 men with UI were unchanged (59.5%) 1/42 men with UI had worsened continence (2.4%)	* Compliance: + 14.1 mL/cmH20 (*p* < 0.01) * Detrusor overactivity: present in 11 of 25 men (−28%) * Mean Pdet at filling: −21.3 cmH20 (*p* < 0.01) * Cystometric capacity: 0.2 mL (*p* > 0.05) * Maximum urethral closing pressure:−16.9 cmH20 (*p* < 0.01)	NR	NR	NR	NR	NR	
Stanskin et al. (1988) [18]	Definition of success: * UI improved or resolved: 2/6 (33%)	10/36 men had UUI (28%) 6/30 men developed de novo UI (20%) 4/6 men with preop UUI were unchanged (67%) 2/6 men with preop overflow UI became continent (33%)	NR	NR	NR	NR	NR	NR	
Koyanagi et al. (1987) [19]	Definition of success: * Voiding without further intervention 86.5% (77/89)	1/89 developed de novo SUI (1.2%) due to sphincter injury	PVR: −35.2 mL (*p* < 0.05) -Flow rate: +15 mL/s (0.05) -VUR was resolved in 15/23 ureters (65%), unchanged in 4/23(17%), and improved in 3/23 (13%) -Compliance: + 22.5 mL/cmH20 (*p* < 0.05) * -Detrusor overactivity: diagnosed in 15 of 26 men and improved or resolved in 13/15. -Mean Pdet at filling: −16.2 cmH20 (*p* < 0.05) -Maximum urethral closing pressure:−5.0 cmH20 (*p* > 0.05) * Data on 26/81 patients only.	NR	NR	NR	1/89 VUR upgrade	12/89 men required further treatment. 5/12 (41%) repeat TURP or Y-V plasty	Same group as Shinno et al.
Koyanagi et al. (1981) [20]	Definition of success: * Patient rendered catheter-free 46/50 (92.0%)	NR	PVR: −20 mL -Flow rate: +7 mL/s -Voided volume: +140 mL	NR	NR	Patient rendered catheter-free 46/50 (92.0%)	NR	NR	
Beck et al. (1994) [21]	NR	12/20 (60%) urge incontinence	NR	NR	NR	NR	NR	NR	
Hou et al. (2017) [25]	Definition of success: * Voiding improvement—reduction of AUR episodes at 12 months after TUR-P: −33% vs.−26.3% (*p* < 0.001) * Reduction of UTI episodes at 12 months after TUR-P: −4.9% vs.−3.3% (*p* = 0.002) * Comparison between ischaemic vs. hemorragic CVA: AUR episodes at 12 months: −32.3% vs. −36.7% UTI episodes at 12 months: −5.6% vs. 0%	NR	NR	NR	NR	NR	Perioperative mortality: 6/577 (1%) vs. 7/6048 (0.1%) (*p* = 0.001)	Re-do prostate surgery 24/577 (4.2%) vs. 422/6048 (7.0%), *p* = 0.061	
Han et al. (2014) [26]	Definition of success: * No clear definition but assessed the postoperativeuse of medications. CVA men had an odds ratio of 5.932 (*p* = 0.001) to use LUTS/BPH medications postoperatively	NR	NR	NR	NR	NR	NR	NR	
Lum et al. (1982) [22]	Definition of success: * Improvement of symptoms: 17/34 (50.0%)	17/34 (50%) incontinence post-OP	NR	NR	NR	NR	NR	NR	
Elsaesser and Stoephasius (1972) [23]	Definition of success: * Outcome defined as good, improved, or not improved based on urine bacterial growth and PVR Good: 24/35 (68.6%) Improved: 4/35 (11.4%) Not improved: 7/35 (20%)	NR	NR	NR	NR	NR	1/35 (3%) death due to urosepsis	2/35 (6%) needed ReTUR-P	
Chang et al. (2022) [24]	Definition of success: * Improvement of symptoms Storage symptoms: frequency and urgency improved in 1/11 (9%) Urge incontinence: no improvement	0/11 men with Parkinson’s disease and UI restored continence (0%)	**Parkinson’s** -PVR: −24.8 mL (*p* = 0.005) -Flow rate: +1.7 mL/s (*p* = 0.511) -Voided volume: −19 mL (*p* = 0.514) -Voided efficiency: +0.11 (*p* = 0.04)	NR	NR	NR	NR	NR	
Chang et al. (2022) [24]	Definition of success: * Improvement of symptoms Voiding symptoms: urinary retention improved from 18 to 2 men (*p* < 0.001), dysuria from 37 to 13 men (*p* < 0.001) Storage symptoms: no change	9/44 men with CVA had worsened continence from 22/50 to 31/44 0/11 men with Parkinson’s disease and UI restored continence (0%) 1/8 men with dementia had worsened continence (12.5%)	**CVA** -PVR: −63.4 mL (*p* = 0.044) -Flow rate: +1.88 mL/s (*p* = 0.122) -Voided volume: +12 mL (*p* = 0.677) -Voided efficiency: + 0.18 (*p* = 0.004)	NR	NR	NR	NR	NR	

# Refers to the 9 men who voided spontaneously.

## Data Availability

Data are contained within the article and Supplementary Materials.

## Supplementary Materials

The following supporting information can be downloaded at: https://www.mdpi.com/article/10.3390/jcm13195846/s1, Supplementary Material S1: Literature Search Strategy.
