# Supplementary material for: Does Surgical Treatment for Benign Prostate Enlargement (BPE)-Related Bladder Outlet Obstruction (BOO) Benefit Patients with Central Nervous System Diseases? A Systematic Review
Source: J Clin Med. 2024 Sep 30;13(19):5846. doi: 10.3390/jcm13195846 (PMC11477414; doi:10.3390/jcm13195846)
Supplement: Supplementary file 1 [file jcm-13-05846-s001.zip › jcm-3200811-supplementary.pdf]

Database: EBM Reviews - Cochrane Central Register of Controlled Trials <June 2023>, Embase <1974 to 2023 July 19>, OVID Medline Epub Ahead of Print, In-Process & Other Non-Indexed Citations, Ovid MEDLINE(R) Daily and Ovid MEDLINE(R) 1946 to Present

Search Strategy:

1 (neurogen* or neuropathic or neurologic* or neuro-urological).tw. (1132834)

2 exp *Nervous System Diseases/ or exp *cerebrovascular disease/ or exp *Cerebrovascular Disorders/ or exp *cerebrovascular accident/ (5070954)

3 exp *spinal cord disease/ or exp *Spinal Cord Diseases/ or exp *Spinal Diseases/ or exp *Alzheimer Disease/ or exp *meningomyelocele/ or exp *multiple sclerosis/ (989360)

4 exp *Parkinson disease/ or exp *meningomyelocele/ or exp *meningocele/ (202522)

5 Diabetic Neuropathies/ or ((diabetes or diabetic) adj3 (neuropath* or cystopath* or urolog*)).tw. (58536)

6 exp *spinal dysraphism/ or ((spina* adj cord) or spina bifida or spinal dysraphism* or cleft spine* or open spine*).tw. (388562)

7 (multiple sclerosis or Parkinson* or Alzheimer* or myelitis or multiple system* atroph*).tw. (999052)

8 (dementia* or progressive supranuclear palsy or corticobasal degeneration or mental retardation or cerebral palsy).tw. (488556)

9 ((cerebral vascular or nervous system or cerebrovascular) adj (disease* or disorder* or accident* or insult)).tw. (117661)

10 (meningocele or meningomyelocele or myelomeningocele* or myelomeningocoele* or myelodysplastic or meningitis).tw. (199311)

11 (Stroke or strokes or poststroke or cerebral tum?r* or brain tum?r* or trauma*).tw. (1981517)

12 (nerve tube defect* or lumbar spine Degenerative disease* or disk prolapse or disk hernia).tw. (1263)

13 (lumbar canal stenosis or cauda syndrome* or hydrocephalus or encephalitis).tw. (168805)

14 (Iatrogenic pelvic nerve lesion* or peripheral neuropath* or tethered cord).tw. (73659)

15 (paraplegia or tetraplegia or hemiplegia or alcoholism or prolapsed disk or prolapsed discs).tw. (128018)

16 Alcoholism/ (217536)

17 or/1-16 (8110251)

18 exp Prostatectomy/ (107947)

19 prostatectom*.tw. (97690)

20 ((prostate or prostatic) adj5 (resect* or surger* or surgical or operat* or incision or TURP or TUIP or TUVP or vaporization or vaporisation or enucleation or laser or "HoLEP" or ThuLEP or BiLEP or PVP)).tw. (42346)

21 ((prostate or prostatic) adj5 (emerging or Urolift or Rezum or Aquabeam or iTIND or embolization or embolisation or laparascop* or robot*)).tw. (5736)

22 prostate/ and (exp surgery/ or exp Urologic Surgical Procedures/ or exp General Surgery/) (24781)

23 or/18-22 (162906)

24 17 and 23 (5406)

25 conference abstract.pt. or Congresses as Topic/ or Conference Review.pt. (4969528)

26 24 not 25 (3723)

27 limit 26 to english language (3128)

28 (animals/ or animal/) not (humans/ or human/) (6305699)

29 27 not 28 (3062)

30 case report/ or case reports/ or (case report or a case or a rare case).ti. (5719846)

31 29 not 30 (2372)

32 note/ or editorial/ or letter/ or Comment/ or news/ or (note or editorial or letter or Comment or news).pt. (5454659)

33 31 not 32 (2304)

34 remove duplicates from 33 (1548)
